# Mapping simulated visual field defects with movie-viewing pupil perimetry

**DOI:** 10.1007/s00417-024-06733-1

**Published:** 2025-01-09

**Authors:** Yuqing Cai, Christoph Strauch, Stefan Van der Stigchel, Antonia F. Ten Brink, Frans W. Cornelissen, Marnix Naber

**Affiliations:** 1 Experimental Psychology, Faculty of Social Sciences, Helmholtz Institute, Utrecht University, Utrecht, The Netherlands; 2 Laboratory for Experimental Ophthalmology, University Medical Center Groningen, University of Groningen, Groningen, The Netherlands

**Keywords:** Pupil perimetry, Simulated visual field defects, Hemianopia, Glaucoma, Modeling pupil size

## Abstract

**Purpose:**

Assessing the quality of the visual field is important for the diagnosis of ophthalmic and neurological diseases and, consequently, for rehabilitation. Visual field defects (VFDs) are typically assessed using standard automated perimetry (SAP). However, SAP requires participants to understand instructions, maintain fixation and sustained attention, and provide overt responses. These aspects make SAP less suitable for very young or cognitively impaired populations. Here we investigate the feasibility of a new and less demanding form of perimetry. This method assesses visual sensitivity based on pupil responses while performing the perhaps simplest task imaginable: watching movies.

**Method:**

We analyzed an existing dataset, with healthy participants (n = 70) freely watching movies with or without gaze-contingent simulated VFDs, either hemianopia (left- or right-sided) or glaucoma (large nasal arc, small nasal arc, and tunnel vision). Meanwhile, their gaze and pupil size were recorded. Using a recently published toolbox (Open-DPSM), we modeled the relative contribution of visual events to the pupil responses to indicate relative visual sensitivity across the visual field and to dissociate between conditions with and without simulated VFDs.

**Result:**

Conditions with and without simulated VFDs could be dissociated, with an *AUC* ranging from 0.85 to 0.97, depending on the specific simulated VFD condition. In addition, the dissociation was better when including more movies in the modeling but the model with as few movies as 10 movies was sufficient for a good classification (AUC ranging from 0.84 to 0.96).

**Conclusion:**

Movie-viewing pupil perimetry is promising in providing complementary information for the diagnosis of VFDs, especially for those who are unable to perform conventional perimetry.

**Supplementary Information:**

The online version contains supplementary material available at 10.1007/s00417-024-06733-1.

## Key messages

*****What is known?***:**

- Pupil perimetry is an objective perimetric technique that can assess visual field defects (VFDs) without overt responses.

*****What is new?***:**

- A new form of pupil perimetry, movie-watching pupil perimetry, can discriminate between conditions with and without simulated visual field defects (sVFD) with an AUC between 0.85 and 0.97
- The detection of sVFD improved when including more movies but 10-20 movies were sufficient for a good VFD classification
- The convenience of movie-viewing pupil perimetry, with unrestricted fixation and low cognitive load, makes it a promising method for assessing VFDs in populations unable to perform conventional perimetry

**Supplementary Information:**

The online version contains supplementary material available at 10.1007/s00417-024-06733-1.

## Introduction

Early detection and precise measurements of visual field defects (VFDs) are crucial for the diagnosis and management of ophthalmological and neurological diseases [25]. Standard automated perimetry (SAP), such as implemented in the Humphrey Field Analyzer, is the conventional technique adopted in clinical practice. SAP maps contrast sensitivity across the visual field by asking the patient to report on presented visual stimuli, such as faint lights, occurring at different locations across 10° to 30° nasally and temporally relative to the centrally maintained fixation. The resulting maps can delineate defective from intact regions by visualizing visual sensitivity thresholds across the visual field [2, 41]. Although SAP has long been the gold standard for assessing VFD, it has several disadvantages: (1) fixation needs to be maintained, but is not controlled for by some perimeters; (2) some patients cannot inhibit saccades or compensatory eye movements [34],(3) SAP is cognitively demanding, especially for children or participants with brain injury [20, 30],(4) as reports are subjective, patients can manipulate test outcomes for economic reasons or due to psychological illness [1],and (5) the sensitivity and test–retest reliability have been questioned [3, 26]. 

To overcome the limitations of conventional SAP, alternative VFD tests have been developed. Such alternative tests are based on measures of psycho- or neuro-physiological indicators of visual sensitivity such as visual evoked potentials (VEP), abnormal oculomotor metrics, or the pupillary light response. One main characteristic of these alternatives is that they do not require patients to manually respond to (dis)appearance of stimuli with a button press. Some of these alternatives still required the patients to respond to the stimuli ocularly, such as tracking a moving target [23, 24], and making saccades to targets [32, 39, 42, 56], while others only require the patients to passively view a fixation point [13, 35, 45, 49] or watch dynamic stimuli such as movies and pictures [5, 21, 22, 28]. Here, we focus on pupil perimetry, an objective perimetric technique that maps intact and defective areas of the visual field by presenting bright stimuli across the visual field and evaluating properties of the pupillary light response (e.g., amplitude) to these stimuli [51, 54, 60]. Pupil perimetry has been used successfully to assess visual field impairments originating from the eye, including retinotopic and optic diseases such as glaucoma and age-related macular degeneration [9, 10, 14, 15, 30, 35, 49], but also impairments originating at the level of the brain [40, 44, 46, 52]. Many efforts have been made over the past decades to improve the comfort, sensitivity, and specificity of pupil perimetry [11, 12, 43–48, 54, 58]. Nevertheless, the current state-of-the-art pupil perimetry techniques, like other objective perimetric tests, still require at least some instruction and are still cognitively demanding, as the participants are required to engage with monotonous and repetitive stimuli.

In this study, we tested a more convenient approach to pupil perimetry using naturalistic, engaging, and dynamic stimuli, namely movie clips. The only task for the participants is to freely watch movie clips. Hence, the method overcomes several limitations of previous perimetry methods, as the assessment is convenient and objective, and the attention of observers is easily maintained with minimal cognitive demands. To assess the feasibility of using pupil size changes during movie viewing to identify VFDs, we analyzed a dataset of participants who freely watched movie clips with or without simulated VFDs (sVFDs) [21]. sVFDs of different shapes and sizes were simulated by overlaying gaze-contingent grey masks on the movie. Pupil size changes over time were modeled with local visual events in the movies (e.g., the sudden appearance of bright objects), using our recent open toolbox (Open-DPSM, [8]). Open-DPSM allows to estimate the relative contribution of visual events across the visual field to pupil size change—and thereby index visual sensitivity. We expected that sVFD and control conditions could be dissociated by evaluating the differences in the contributions of visual events to pupil size changes in masked and unmasked regions.

## Methods

### Dataset

We used an existing open-source dataset from Gestefeld et al. [21]. The original data and stimulus materials (movies and masks used to simulate VFDs) can be retrieved from DataVerseNL (https://doi.org/10.34894/LEYVL8). The central properties of the dataset are described below, see the original publication for full details.

### Participants

The dataset contains data from 70 healthy participants (*M*_*age*_ = 20*, SD*_*age*_ = 3.3 years; 60 females, 10 males). All participants had normal or corrected-to-normal visual acuity and no visual field defect. Visual acuity was tested with the letter chart of Early Treatment of Diabetic Retinopathy Study (EDTRS) and their visual field was tested using Frequency Doubling Technology (Humphrey FDT model 710, C-20–1 program; Carl Zeiss Meditech, Jena, Germany)”. The study was approved by the ethics committee of the Department of Psychology of the University of Groningen.

### Experiment setup

Participants watched 94 movie clips of one minute each with (1) simulated hemianopia (left or right; n = 40) or (2) simulated glaucomatous visual field defects (large nasal arc, small nasal arc, and tunnel vision; n = 30), and each with a control condition without simulated VFD. For the simulated hemianopia, participants watched either 20 or 74 movies for sVFD (either left or right hemianopia was tested) and control conditions. For the simulated glaucoma, participants watched 10 to 22 movies for sVFD (all three were tested) and control conditions. The reason why not all the participants watched the same number of movies per condition was not addressed in the original paper.

Eye movements were recorded with an EyeLink 1000 eye tracker (SR research). Simulated VFDs were created by superimposing mid-level brightness, grey, opaque masks (i.e., a pixel of such a mask has a luminance value of 128) onto the movie clips to mimic the VFDs in real patients. The position of the mask was synchronized with gaze (i.e., gaze-contingent) to always cover the same part of the visual field in a retinotopic coordinate system. For instance, if the participants looked at the right-most side of the screen in a left hemianopia condition, the entire screen would be masked.

## Data analysis

### Modeling pupil size changes

**Fig. 1:**
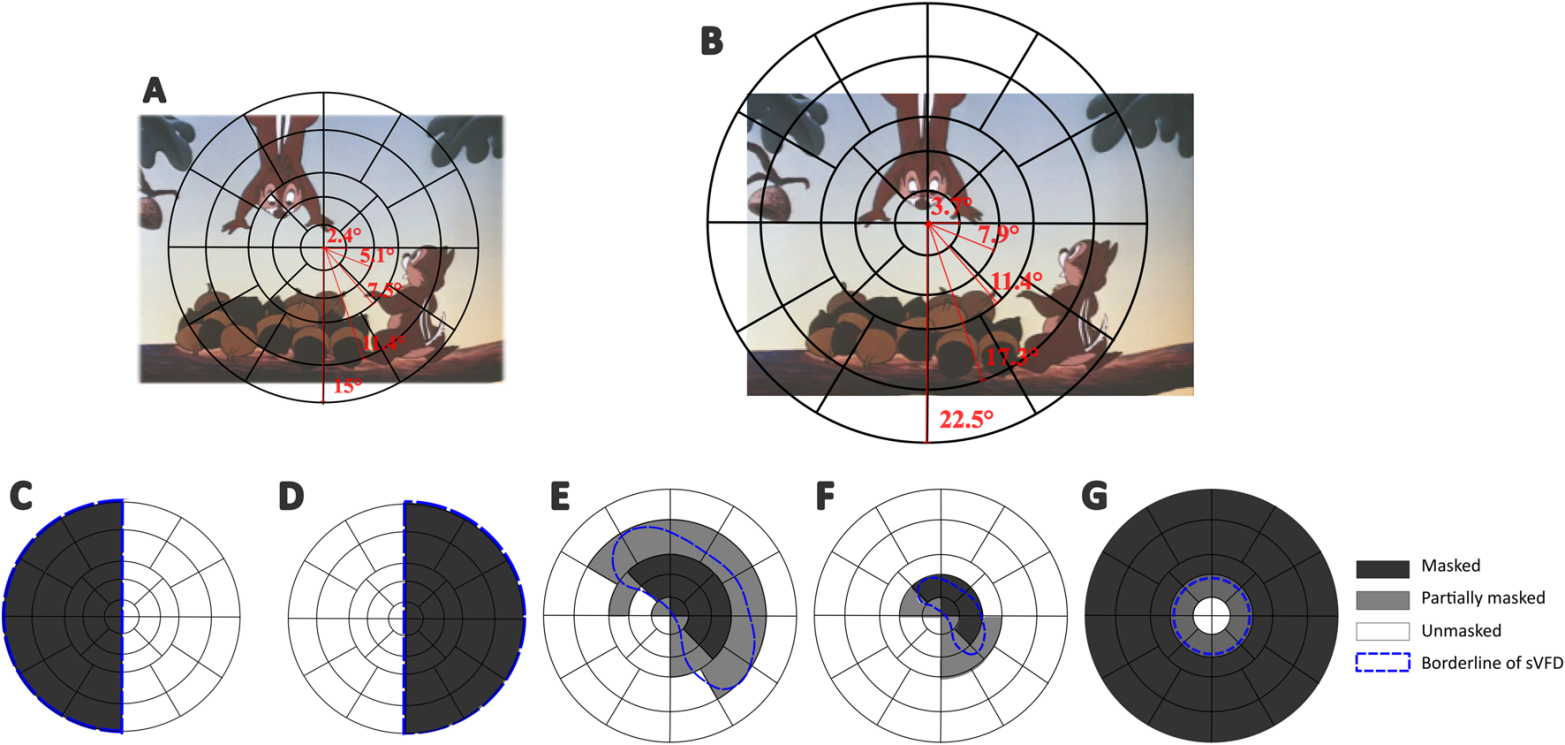
Visual field regions overlaid on a movie frame and different simulated visual field defects (sVFDs). **A** The visual field was divided into separate regions in the form of five eccentricities (marked with black lines) for Experiment 1 (simulated hemianopia), which was based on standards in the field of pupil perimetry (e.g. [7, 45, 49, 58]). **B** Same plot as in **A**, but for Experiment 2 (simulated glaucoma) during which movies were displayed on a larger monitor. This example, where the visual field is shifted towards the left of the movie, illustrates the gaze-contingent adaptation of the visual field that moved along with the gaze position. **C-D** Simulated hemianopia on the left and right side. The dark grey regions represent the masked areas, the white regions the unmasked areas, and the blue dashed lines the outlines of the mask; **E–G** Same as panel **C-D**, but corresponding to simulated scotomas: large nasal arc (**E**), small nasal arc (**F**), and tunnel vision (**G**). Some regions were partially masked. Regions were classified as masked instead of unmasked when the mask exceeded 50% of the region

With an open-source toolbox “Open-DPSM” (Open Dynamic Pupil Size Modeling; [8]), we predicted the pupil size changes during movie-watching with visual events (i.e., luminance and contrast changes; to both of which pupils respond) that were extracted from the movies. Events were extracted in a gaze-contingent manner for each movie frame to mimic the video image projected onto the retina (see Fig. 1A-B for gaze-contingent adaptation of visual events), that is, the center of the frame image was first re-aligned with the gaze position at each time point and the visual field was divided as regions (see Fig. 1A-B for the division of visual field regions). Visual events were extracted separately for each region.

We expected that visual events would contribute differently to the pupil size change depending on the visual field region, as the visual events within masked regions – invisible due to the simulated gray scotoma – should not influence pupil size (see Fig. 1** C-G** for an illustration of sVFD masks on top of visual field regions). Hence, pupil size changes to visual events in each region were modeled separately first with a response function (see supplementary material for more information). To identify the regions where visual events had a greater impact on pupil size changes, we weighted predicted pupil size changes by multiplying predicted pupil size changes in each region with a corresponding regional weight. The regional weights were free parameters, and they were determined by optimizing procedure that all possible combination of regional weights were iteratively tested until the best one was found. This combination predicted the observed pupil size change the best that the root mean square error between the predicted and observed pupil size change was minimal. The regional weights can be interpreted as (in)sensitivities across the visual field predicted with recorded pupil size changes, as they capture how strongly visual events affect the observed pupil response depending on the location of the visual event. In other words, the predicted regional weights for the masked regions should be significantly smaller than those for unmasked regions. All the trials in each condition were first combined so that a single set of regional weights was predicted by the model per condition. As the steps of data analysis were identical to those in Open-DPSM, we refer to Cai et al. [8] for more detailed information (a description of the modeling method is also provided in Supplementary Materials Part 1).

### Reconstructing visual field defects and statistical testing

VFDs were reconstructed using the optimal regional weights derived from the model. The weights were visualized as maps per condition, per participant, and averaged across participants. In addition, as the strength of pupillary responses (and thus the weights) to visual events systematically varies between the left versus right, and the top versus bottom visual field locations (i.e. due to visual field anisotropies or asymmetries; [30, 47]), the result of the control condition for each participant was subtracted from each sVFD condition in order to isolate the effect of the sVFDs.

To quantify whether the regional weights differed between masked and unmasked regions in sVFD conditions but not the control condition, we calculated the mean of the regional weights per participant and per condition. Two signal detection estimates, the area under the curve (*AUC*) of the receiver operating characteristics (ROC) and *d’*, the distance between the peaks of the distributions in the z-scored regional weights, provide a quantitative measurement of the discriminative power between unmasked and masked regions in the sVFD conditions, and between the sVFD versus control conditions. Specifically, we expected that the weights could dissociate (1) masked from unmasked regions in sVFD conditions since unmasked regions should obtain much higher weights than the masked regions; and (2) sVFD from the control conditions since the differences between the regional weights assigned to the masked and unmasked regions should be much larger in sVFD than in the control condition. To better compare our results to those of previous studies, we further report classification accuracies at optimal cut-off points as determined through grid search [19], which meant iteratively searching all possible cut-offs until the best was found.

## Results

### Model performance

Before evaluating the discriminative power of movie-viewing pupil perimetry in dissociating between the sVFD and control conditions, we first confirmed that pupil size changes to visual events could be modeled successfully and the model performance in all conditions was assessed with R^2^ and RMSE (see Table 1 for a summary).

**Table 1:** Mean model performances across all conditions

			R^2^	RMSE
Simulated hemianopia	Control		0.28	0.84
	Left hemianopia		0.14	0.92
	Right hemianopia		0.13	0.93
Simulated glaucoma	Control		0.30	0.83
	Large nasal arc		0.22	0.88
	Small nasal arc		0.26	0.85
	Tunnel vision		0.18	0.89

Abbreviation: RMSE = root mean squared error

Note that the sVFD condition resulted in generally smaller explained variances than the control condition. This was to be expected as fewer visual events were presented in the movies with sVFD, resulting in less variance that could be explained.

### Reconstruction of visual field defects maps

**Fig. 2:**
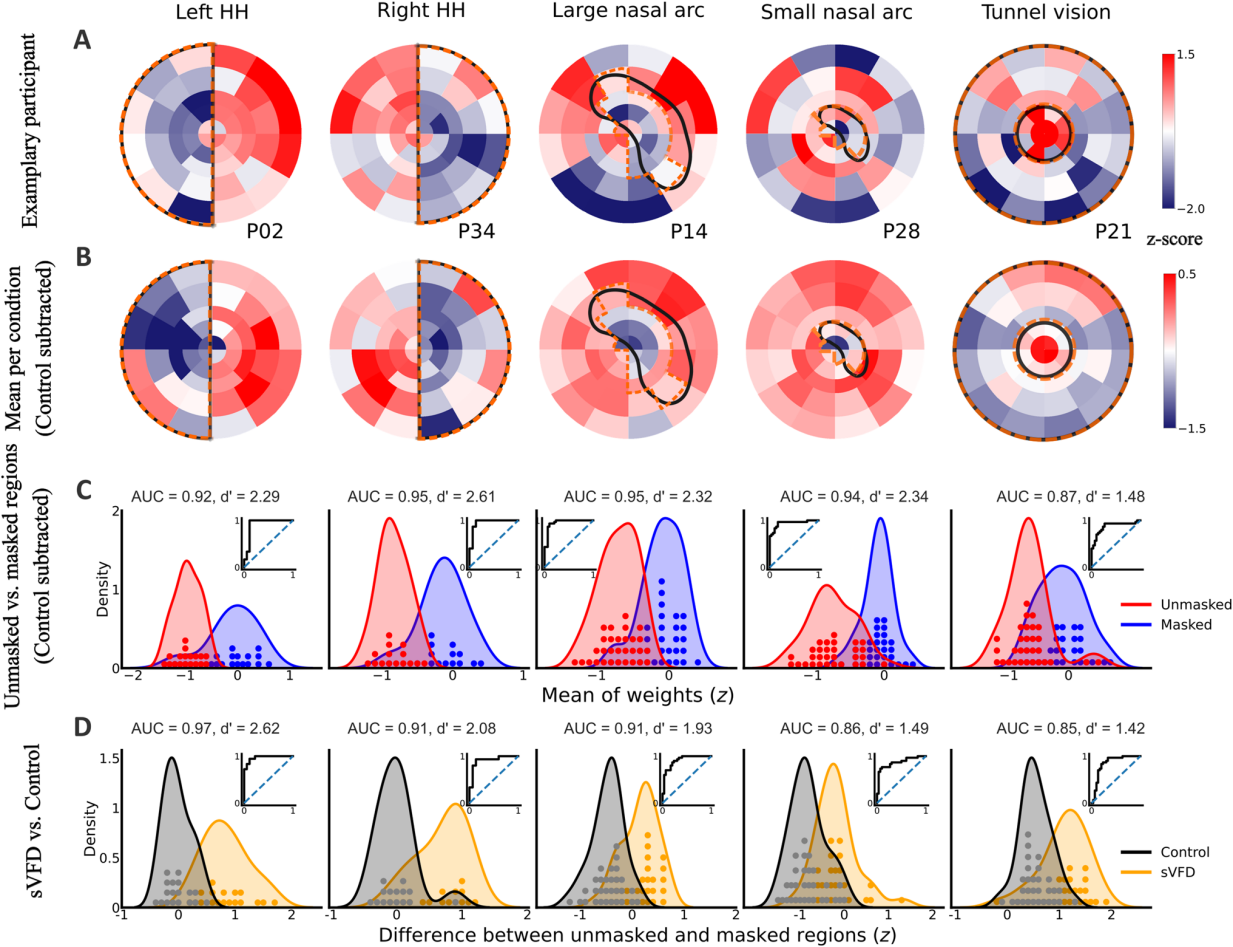
Modelled regional weights (red = high; blue = low) for **A** one exemplary participant per simulated visual field defect (sVFD) condition; **B** Mean weights of all participants per sVFD condition after subtracting weights of the control condition; **C** Distribution of weights (z-standardized) in unmasked regions (in red) and masked regions (in blue) per sVFD condition. ROC curves are shown as subplots; **D** Distribution of the average weight difference between unmasked and masked regions (z-standardized) per sVFD condition (orange), compared with the control condition (grey). Each dot indicates one participant. Positive scores on the x-axis mean that masked regions evoked weaker pupil responses than unmasked regions

From the modeling results of Open-DPSM, we obtained the best-fitted regional weights, which were used to visualize visual sensitivity across different regions. Masked regions should be assigned with much lower weights than unmasked regions. First, we reconstructed each map of all sVFDs per participant (see examples in Fig. 2A). Here, the patterns of the sVFD already emerged, and the exact shapes became even clearer after averaging across participants (see Fig. 2B).

### Differences in regional weights between unmasked and masked regions

To quantify whether regional weights could reveal the visibility of the regions, average weights of unmasked and masked regions were compared per sVFD (see distribution of average weights across all participants in Fig. 2C). In all conditions, the regional weights for unmasked regions were higher than those for masked regions, with an average *AUC* of 0.94 and 0.92, an effect size *d’* of 2.45 and 2.04, and an average classification accuracy of 0.93 and 0.88 for simulated hemianopia and simulated glaucoma, respectively (see Fig. 2C for the results of *AUC* and *d’* in each condition). These results show that the regional weights can dissociate unmasked from masked regions above the chance level.

### Sensitivity and accuracy of regional weights in separating sVFD and control conditions

Similarly, we evaluated the performance of regional weights in classifying sVFD and control conditions using the difference in regional weights between masked and unmasked regions. The masked and unmasked regions in the control condition were defined using the same regions in each sVFD condition. As the “masked” regions in the control condition were not actually masked, the regional weight differences should be much lower than in sVFD condition. The distribution of regional weight differences between unmasked and masked regions is graphically represented alongside the corresponding control condition in Fig. 2D**.** The weight distributions for sVFD conditions and control conditions were all highly distinct, with an average *AUC* of 0.94 and 0.87, an average effect size *d’* of 2.35 and 1.61, and an average classification accuracy of 0.90 and 0.83 for simulated hemianopia and simulated glaucoma respectively (see Fig. 2D for the results of *AUC* and *d’* per condition). The results for simulated hemianopia and the large nasal arc were descriptively better than the results for the small nasal arc and tunnel vision. Across all sVFD conditions, the worst *AUC* results were obtained for tunnel vision (*AUC* = 0.85) and the worst *d’* for the small nasal arc (*d'* = 1.66). These results show that Open-DPSM can dissociate sVFD conditions from the control condition.

In addition, as different conditions contained different numbers of movies, we also tested whether more data was associated with better detection of sVFDs. To test this, we reran the model with subsets of movies for participants with enough data (see Supplementary Materials Part 2 for details). The results suggested that adding data generally improves results, although the improvements varied among conditions. In addition, the accuracy in classifying sVFD from controls was already good for 10- and 20-movie models (10-movie model: AUC ranging from 0.84 to 0.96, d’ ranging from 1.42 to 2.28, accuracy ranging from 0.82 to 0.94; 20-movie model: AUC ranging from 0.80 to 0.96, d’ ranging from 1.04 to 2.08, accuracy ranging from 0.85 to 0.93, see Supplementary Fig. 2 for details).

Apart from the modeling method reported here, we also applied an event-related pupillary response method to extract the amplitudes of the pupillary response to visual events occurring in the masked and unmasked regions directly from raw pupil data. The results indicated that simple event-related pupil responses to visual events can achieve above-chance dissociation of masked versus unmasked regions, but the dissociation was much worse than the modeling method. The details of the approach are reported in Supplementary Materials Part 3 to facilitate the readers’ understanding of the modeling method as they are essentially based on the same idea.

## Discussion

The current study demonstrates the feasibility of movie-viewing pupil perimetry in dissociating masked from unmasked regions of the visual field. The relative strengths of pupil responses to luminance and contrast changes in different regions were obtained by modeling the empirically observed pupil size changes. Whether the obtained relative strengths of pupil responses can reconstruct visual sensitivity across the visual field in different conditions was then evaluated. We showed that with dynamic and naturalistic stimuli, the modeling approach could successfully dissociate regions covered by the simulated scotomas from unmasked regions. Overall, it was accurate in detecting both simulated hemianopia (accuracy = 0.9) and simulated glaucoma-like scotomas (accuracy = 0.83). An important advantage of the here-introduced movie-viewing pupil perimetry over previously used pupil perimetry techniques is that it merely requires the participants to freely view movies, which minimizes the need for instructions from examiners and cognitive demands from participants. Because of this convenience, we foresee that this method can be applied particularly in younger populations (under 5 years old) and brain-injured patients, who experience challenges when being assessed with conventional SAP or existing methods for objective perimetry.

Some previous studies have also described perimetric techniques using free viewing of complex stimuli to classify (s)VFDs (e.g., [17, 18]) and map the visual field [21, 22] with eye movement-based approaches. In the current study, we used the data from Gestefeld et al. [21], allowing a direct comparison of the performance of each method. The performance of our pupil perimetry method in classifying sVFD was comparable to the eye movement-based method reported by Gestefeld et al. [21] (an overall accuracy of 0.86 in classifying sVFD was reported in [21]).

To further explore the applicability of the current perimetry, we tested how the amount of data related to modeling results and how much data would be sufficient to detect sVFDs. The results showed that, descriptively, the model generally became better in detecting sVFD when more data was included, but this increase in performance saturated at around 40 min of movie watching per condition (Supplementary Fig. 2). In addition, even ten or twenty minutes of movie watching produced good dissociation between sVFD and unoccluded areas. One further must consider that the model was trained using 70% of the data, meaning that less data would actually be needed to achieve the reported classification in practice. Although the duration of the current perimetry is still longer than the existing gaze-contingent pupil perimetries (e.g. [45]) and standard automated perimetry (usually less than 10 min per eye, e.g., [33]), movie-watching is an entertaining activity that anyone can easily perform so we perceive the current method and duration as already feasible to be applied in practice. Together, this makes the here introduced form of perimetry a promising technique to complement the conventional SAP in testing regular patients, or perhaps as an alternative for patients who cannot perform conventional SAP tasks.

The results of models with the same amount of data across participants (i.e. 10- and 20-movie models) also demonstrated that larger sVFD conditions (tunnel vision, left/right hemianopia) did not outperform the sVFD conditions with smaller defects (small/large nasal arcs), which is different from previous findings of pupil perimetry being generally better at detecting larger visual field defects than smaller ones [15, 40].

The current study focused exclusively on the amplitudes of pupillary responses to luminance and contrast changes in different regions, which allowed the model to account for a limited proportion of variance in pupil size changes (with a median R^2^ = 0.22). Other factors, including other low-level visual events such as spatial frequency, higher-cognitive factors such as arousal and attention, audio, muscular noise, as well as noise introduced by the eye-tracker and high-frequency components of the pupillary signals, can all influence pupil size changes (see [53] for a review). However, those factors are much less meaningful than luminance and contrast changes in terms of testing visual sensitivity and thus were considered irrelevant for the current study. The explained variance achieved here has therefore to be put into perspective with only the contribution of luminance and contrast changes on the pupil rather than all pupil size changes. Despite further room for improvements in overall model performance, the diagnostic properties of our pupil perimetry method were already sufficiently high to detect sVFDs well.

## Future directions

After this proof of concept, the most important next step lies in testing our method on patient data. The rationale behind using sVFDs in healthy participants was to have an established ground truth of the position of a defect, to test and optimize the mapping method. However, it is worth noting that the statistical testing adopted by the current study that compared the sVFD and control conditions *within* participants will be impossible when testing patients with VFDs. Testing patients with VFDs will require comparing each individual patient to an average of healthy controls. Moreover, the nature of VFDs in patients may result in distinct changes in pupil response and may pose several potential challenges when applying the method. For instance, in hemianopia patients, reduced pupil light responses to events in defected regions are found, but these responses are not fully reduced, and the degree of reduction varies across individuals [50]. In addition, patients with VFDs often do not perceive their defects as completely blank regions [16] as the VFDs are sometimes perceptually filled in with the visual patterns of the regions surrounding the VFDs [27]. It is unknown how such subjective filling-in phenomena influence pupil size changes. Further investigation is also needed to explore how well the sensitivity of dynamic and continuous stimuli in detecting sVFD extends to patients. Previous studies have tested the applicability of pupil perimetry in patients with different origins of VFD, such as in cerebral visual impairments [40, 44, 46], and glaucoma [4, 9, 10, 35, 36, 57], and found comparable diagnostic properties as with SAP. Furthermore, attentional deficits such as spatial neglect might be retrievable via pupil perimetry [55]. Thus, how well movie-viewing pupil perimetry generalizes across these diverse populations remains to be tested.

Beyond changes in luminance and contrast, other visual events eliciting pupil constrictions, such as changes in color, spatial frequency, and orientation [6, 29, 31, 53] can also elicit pupillary responses (see [8] for further ideas on improving Open-DPSM). All those factors were regarded as unexplained noise in the current model. The fact that the current model was already fully capable of distinguishing sVFDs from controls shows the high potential of detecting sVFDs even better when pupil modeling methods advance further. Moreover, the degree of impairments in pupillary response to those features may differ across different etiologies of VFD. For example, retinal damage (e.g. due to glaucoma) and cerebral impairments (e.g. hemianopia) may lead to different altered pupillary responses to chromatic changes [9, 37]. In addition to pupillary response amplitude, other indicators, such as the velocity of the pupil response, may be impacted by a VFD [38, 59]. We therefore see the potential to achieve similarly effective diagnostic properties as the existing perimetry methods by incorporating these further features to advance the here-introduced pupil modeling method, which can be accomplished with the dynamic and content-rich nature of movies as stimuli.

Another important factor to consider for applicability is what stimuli properties may influence model performance the most. However, this question is difficult to test with Open-DPSM directly, as data were combined across movies first, and a single set of weights per participant and condition was predicted (not per movie). However, it is interesting to speculate about which movies should best be used. Based on the current model, factors such as the number or amplitude of luminance changes in the movie, the spread of luminance changes across regions of the visual field, the multicollinearity of luminance changes among regions, and the number of other types of visual events such as color and spatial frequency, are all plausible factors that could influence model performance. Future work could therefore seek to optimize the technique by investigating stimulus properties.

Given that both eye movement perimetry [21] and pupil perimetry are based on eye-tracking data, another plausible future direction is to integrate the two approaches to improve signal detection. The two approaches may operate on different underlying mechanisms and identify qualitatively different aspects of VFDs. For instance, Gestefeld et al. [21] reported that the effectiveness of viewing priority in identifying hemianopia may be related to viewing strategies in simulated hemianopia and also in patients. In contrast, viewing strategies are irrelevant for pupil perimetry. A synergistic perspective of the two approaches would likely improve the signal-to-noise (and thus diagnostic properties) and result in more specific and sensitive visual field maps.

## Conclusion

The current study demonstrates the feasibility of detecting simulated VFDs using movie-viewing pupil perimetry. By reconstructing visual field sensitivities using an open-source toolbox that models dynamic pupil size responses over time, we showed how different visual field locations contribute to these dynamics. This is the first endeavor to extend pupil perimetry towards more dynamic and engaging stimuli. It is an important step towards expanding the applicability of pupil perimetry in the assessment of VFD in patients who have difficulty with conventional SAP, such as very young children and cognitively impaired patients.

## Supplementary Information

Below is the link to the electronic supplementary material.

Supplementary file1 (DOCX 1276 KB)

## Data Availability

All data are available via DataVerseNL 10.34894/LEYVL8

## Code availability

The modeling code is available at https://github.com/caiyuqing/Open-DPSM

## Declarations

### Preregistration

The experiment was not preregistered.
