# Supplementary material for: Mapping simulated visual field defects with movie-viewing pupil perimetry
Source: Graefes Arch Clin Exp Ophthalmol. 2025 Jan 9;263(6):1641–50. doi: 10.1007/s00417-024-06733-1 (PMC12238215; doi:10.1007/s00417-024-06733-1)
Supplement: Supplementary file 1 — Supplementary file1 (DOCX 1276 KB) [file 417_2024_6733_MOESM1_ESM.docx]

Supplementary materials to “Mapping simulated visual field defects with movie-viewing pupil perimetry”

# Part1: Open-DPSM modeling method

## Methods

### Preprocessing of eye-tracking data

For each movie (trial), pupil size data was preprocessed as follows: First, instances where the rate of change of pupil size exceeded three standard deviations above the mean, were identified as blink onsets and offsets. Those blink periods were subsequently removed from the data, and the deleted blink periods of the pupil and gaze data were interpolated. The eye tracking data were then down-sampled to match the sampling rate of the movie (25 Hz), ensuring that each movie frame had corresponding unique pupil size and gaze position data. Following down-sampling, pupil and gaze data were z-standardized over time by subtracting the mean and subsequently dividing by the standard deviation over time per movie clip.

### Visual field regions

To map the contributions of visual field regions to pupil responses (i.e., sensitivities), the visual field was divided into separate regions, and visual events were extracted individually for each region. In Cai et al. (2023), the regions were rectangular in shape. Here, we optimized the shape by using a circular shape and divided the visual field into 44 regions across five eccentricity rings, based on standards in the field of pupil perimetry (Bell et al., 2010; Portengen et al., 2022; Sabeti et al., 2017; Wilhelm et al., 2000). The map covered a 30- or 45-degree horizontal and vertical field of vision for Experiments 1 and 2, respectively.

### Gaze-centered visual events extraction

As the participants freely viewed the movies, shifts in gaze position resulted in corresponding shifts in the position of the video image projected onto the retina. For example, if gaze shifted toward the top left of the screen, the center of the retina would not correspond to the center of the screen. As the background surrounding the monitor was black, we embedded the movie frames in a larger black rectangle to simulate what really fell on the retina of the participants. At each time point, the center of the circular regions was re-aligned with the gaze position, which was defined as the center of the visual field.

Visual events (i.e. luminance changes and contrast changes) were extracted for each region independently. Luminance changes in each region were calculated as the difference in luminance, averaged over all pixels in a corresponding region, between consecutive frames. Contrast changes were approximated by the absolute value of the luminance changes.

### Modeling the pupil size change to visual events

We adopted the convolutional model (Open-DPSM) to model the continuous pupil size changes to all luminance and contrast changes and thereby obtain the relative contributions of visual events to pupil size changes in each visual field region, as described before. The convolutional modeling method approximates pupillary responses to visual events with a prototypical pupil response to stimulation, a so-called response function (**Supplementary Fig. 1A**). Two unified response functions were convolved with luminance changes and contrast changes (**Supplementary Fig. 1B**) in all regions respectively. The predicted pupil size change was modeled as the sum of all the responses at each time point (**Supplementary Fig. 1C**, green line). The shape of the two response functions was controlled by two free parameters respectively, and an additional free parameter was introduced to control for the relative amplitude of the peak for contrast response compared with luminance response. Note that contrast responses produce temporary (transient) responses that go back to baseline within several seconds, while luminance changes elicit sustained responses. Hence, the pupil size change trace modeled for luminance changes (not contrast) was accumulated to produce sustained responses (**Supplementary Fig. 1D**, black line).

To discern the relative contribution of each region to pupil size changes, 44 additional parameters were incorporated as regional weights. Predicted pupil size changes in each region, induced by convolution of luminance and contrast changes with response functions were then multiplied by the respective regional weight (**Supplementary Fig. 1C**). The final predicted pupil size changes across all the regions were computed by averaging all these weighted predictions (see **Supplementary Fig. 1D** an exemplary illustration of the result of combining two regional weights into corresponding modeled pupil size changes). The events within the masked regions should elicit no pupillary response (**Supplementary Fig. 1E**).

In total, our model encompassed 49 free parameters. Nelder-Mead simplex search algorithm was adopted to search through different combinations of parameter values, minimizing the root mean squared error (RMSE) between the predicted and the actual pupil size change. To prevent overfitting, we used cross-validation, dividing all trials in each participant randomly into training (70%) and testing (30%) datasets. Five iterations of cross-validation were performed, and all reported results were the averaged testing results across iterations. To evaluate the model performance, R-squared and RMSE between the predicted and the observed pupil size changes were calculated for each condition (for the full procedure see Cai et al., 2023).


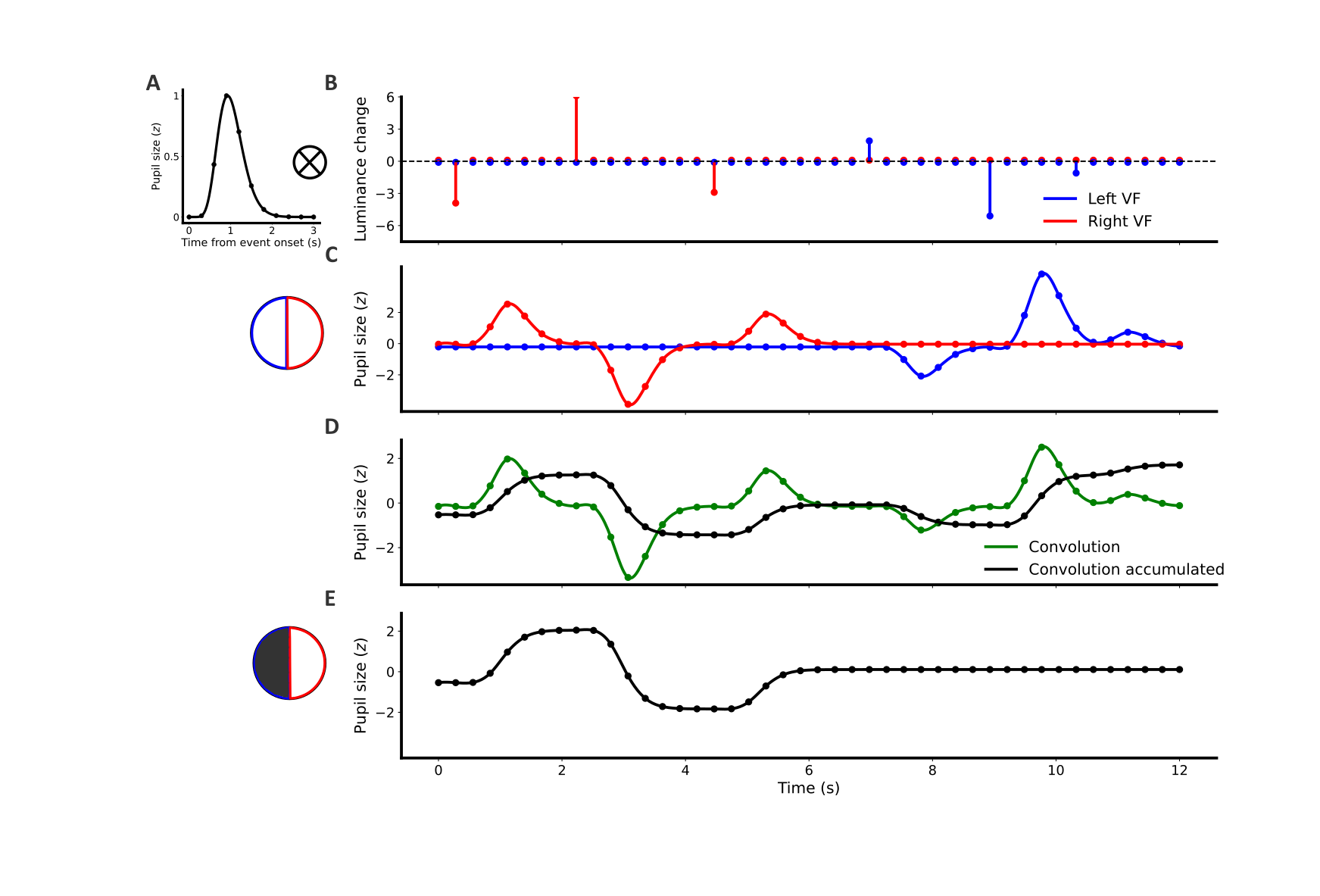


***Supplementary Fig. 1*** An illustration of the convolutional approach of Open-DPSM. **A** Pupil response function used for convolution. **B** Illustrative data of luminance changes over time in left and right visual field (VF) regions (note that more regions are created in the actual analysis; see **Fig. 1A** in the main text), represented by blue and red, respectively. Changes can be either positive (dark to bright) or negative (bright to dark). In this example, luminance changes in the “red” (right) region only occur from 0 to 6 seconds and those in the “blue” (left) region only occur after 6 seconds. **C** Simulated pupil responses by convolving the response function in **A** with luminance changes in **B**, showing predictions of transient changes for each of the two regions. Note that pupillary responses are opposite to the luminance changes as luminance increments induce a constriction. **D** If the two regions are both visually intact (unmasked; sensitive), each should receive a comparable weight, that is, luminance changes in both regions contribute to the pupil size change equivalently. Overall predicted transient responses (green) are calculated by the sum of the two predictions in panel **C**. Additionally, an accumulated (sustained) prediction is also plotted in black, which is the final predicted luminance change by the model. **E** Expected results for a left-sided visual field defect (red = unmasked). The regional weights are modeled as 1 and 0, as only the events in the first 6s (in the unmasked red region) affected pupil responses.

##### Regularization on regional weights

Due to the interrelated nature of the content (and thus luminance changes) across (movie image) regions, visual events within different regions exhibited high correlation (especially for those that were adjacent to one another). As an extension to Open-DPSM (Cai et al., 2023), ridge regression, a regularization technique that suppresses artifacts in weights caused by multicollinearity, was therefore applied to bolster the predictive accuracy of the linear regression model and to mitigate overfitting. Ridge regularization introduces a penalty term into the cost function, discouraging excessively large coefficients for predictors (i.e., regional weights). Notably, only the many parameters associated with regional weights underwent regularization, while the parameters of the response function remained unchanged.

The regularization process was implemented using the "Ridge" class within the "linear" module of the "sklearn" package. The strength of regularization was fine-tuned via the "alpha" parameter. The optimal alpha value was determined by an evaluation of the root mean squared error (RMSE) of testing sets (an alpha resulting in the smaller test RMSE represents a better model fit) and the difference in RMSE between training and testing sets (an alpha resulting in the least difference in RMSE corresponds to a model with minimal overfit).

# Part 2: More data improved model performance in detecting simulated visual field defects

The previously reported model in Part 1 was run with data of all movies in all participants, with different participants/conditions containing different numbers of movies (ranging from 10-74 movies; **Supplementary Fig. 2A**). We expected more data to be associated with better detection of sVFDs. To test this, we reran the model with subsets of movies for participants with enough data. Models with a subset of 10, 20, 40, or 60 movies were tested for simulated hemianopia conditions. As simulated glaucoma conditions had at most 22 movies, only 10- and 20-movie models were tested for this condition. For each model, dissociations between (1) masked and unmasked regions and (2) sVFD and control conditions were evaluated using AUC and d’ measurements, similar to those reported in **Fig.2C & D** in the main text. The results suggested that adding data improves results, and 40 movies were descriptively sufficient for an optimal classification (see **Supplementary Fig. 2B-E**). Note that in the 10- and 20-movie models, the results of simulated hemianopia were no longer better than the results of simulated glaucoma, but the accuracy in classifying sVFD from controls was already good (10-movie model: accuracy = 0.9 for simulated hemianopia and 0.83 for simulated glaucoma; 20-movie model: accuracy = 0.9 for simulated hemianopia and 0.88 for simulated glaucoma).
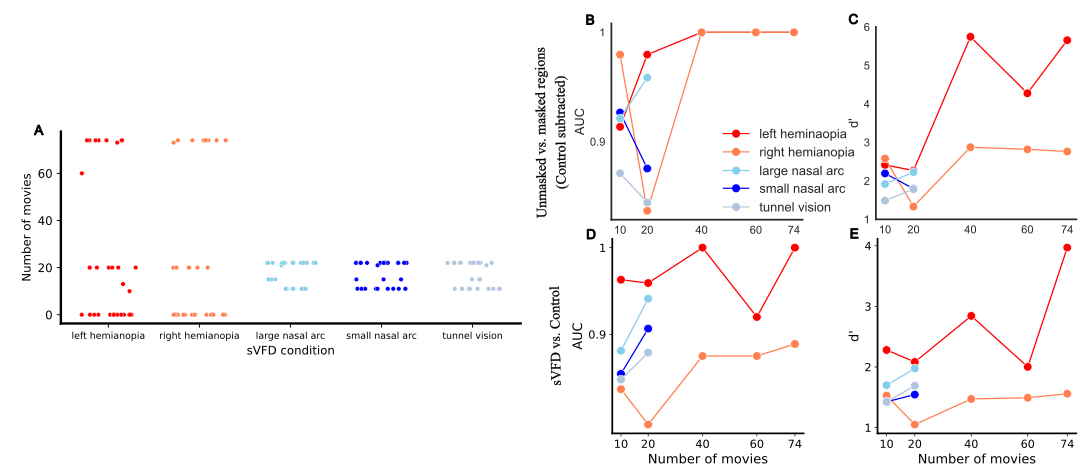


***Supplementary Fig. 2*** **A** The number of movies in five sVFD conditions (data points represent participants in the corresponding condition). Model performance in dissociating **B & C** masked and unmasked regions in sVFD conditions (same AUC and d’ measurements as those in Fig. 2C in the main text) and **D & E** sVFDs and control conditions (same AUC and d’ measurements as those in Fig. 2D in the main text) for models using different numbers of movies. Note that simulated glaucoma conditions do not have results for 40-, 60- and 74-movie models because there were maximally 22 movies per condition here.

# Part 3: Event-related Pupillary Response (ERPR) analysis

As a complementary to the modeling method, we also extracted event-related pupillary responses (ERPRs) to distinguish sVFD from controls.

In the masked regions, visual events that would have happened within those regions if they would not have been masked, could not influence pupillary responses. Therefore, pupil response amplitude between masked and unmasked regions should be different in masked trials (sVFD trials), and therefore could dissociate between sVFD from control trials. As the idea underlying the ERPR method is essentially the same as the modeling method, we reported the ERPR method here only as a simplified illustration of the modeling method.

## Methods

### ERPR extraction

Events of luminance changes in the masked and unmasked regions were determined post-hoc with a threshold (all the luminance changes below ±1 cd/m^2^ were removed). Visual field regions falling only partially in the simulated scotoma were considered as masked regions if 50% or more was occluded by the scotoma. All possible magnitudes of luminance changes were categorized into five separate bins using percentiles (0-20% darkest (bin0), 21-40% dark (bin1), 41-60% weakly dark and bright (bin2); 61-80% bright (bin3), 81-100% brightest (bin4)). Only the strongest and thus most robust luminance changes (bin0 and bin4; i.e., luminance changes of around ±15 cd/m^2^ that lead to robust pupil responses) were selected for the current analysis (see **supplementary Fig. 3 and Fig. 5** for mean magnitudes of events)^[[1]](#footnote-2)^.

With the timing of each event in the masked and unmasked regions as the onset, event-related pupil responses were then extracted by segmenting the pupil time-series data into 3s segments. Baseline correction was performed for each of the ERPRs by subtracting the average pupil size before 250ms. All ERPRs were further z-standardized by dividing the standard deviation of the ERPRs. After extracting all ERPR, the mean of ERPR for bin0 and bin4 was calculated for each participant (see **supplementary Fig. 3 and Fig. 4** for average pupil responses in masked and unmasked regions in sVFD conditions (first row) and control conditions (second row) across all participants).

### *Control-subtracted pupil response*

For sVFD conditions, we expected that pupil responses to luminance changes in the masked regions would be smaller than those in the unmasked regions. For control conditions, the pupil responses in the masked and unmasked regions should be similar (for now ignoring potential hemifield asymmetries). Hence, the ERPRs in the control conditions were subtracted from sVFD conditions for each participant. After control subtraction, pupil response to visual events in unmasked regions should be close to 0 while that to events in masked regions should be larger or smaller than 0, determined by whether the luminance changes were positive (bin4) or negative (bin0). To correct the direction of control-subtracted ERPRs, pupil responses of bin0 were further multiplied by -1 so that control-subtracted ERPRs in masked regions should be positive (see **supplementary Fig. 3 and Fig. 5**, **first row** for control-subtracted ERPRs in unmasked and masked regions for bin0 and bin4 events).

### Statistical testing

To determine whether the ERPRs can distinguish sVFD and control conditions, several statistical tests were adopted. Firstly, the differences between the control-subtracted ERPRs in masked and unmasked regions were tested with paired-sample t-test to quantify whether the control-subtracted pupil responses at each time point were different. Secondly, same signal detection estimates as the modeling methods described in main text, the area under the curve (AUC) of the receiver operating characteristics (ROC) and the d’, were adopted (see the main text “Reconstructing visual field and statistical testing” section for more details). AUC was first calculated for each time point. Overall AUC and d’ between the average control-subtracted ERPRs after 0.5s for the masked and unmasked regions were also calculated. In addition, AUC and d’ were calculated to compare differences in pupil response for unmasked and masked regions between sVFD and control conditions.


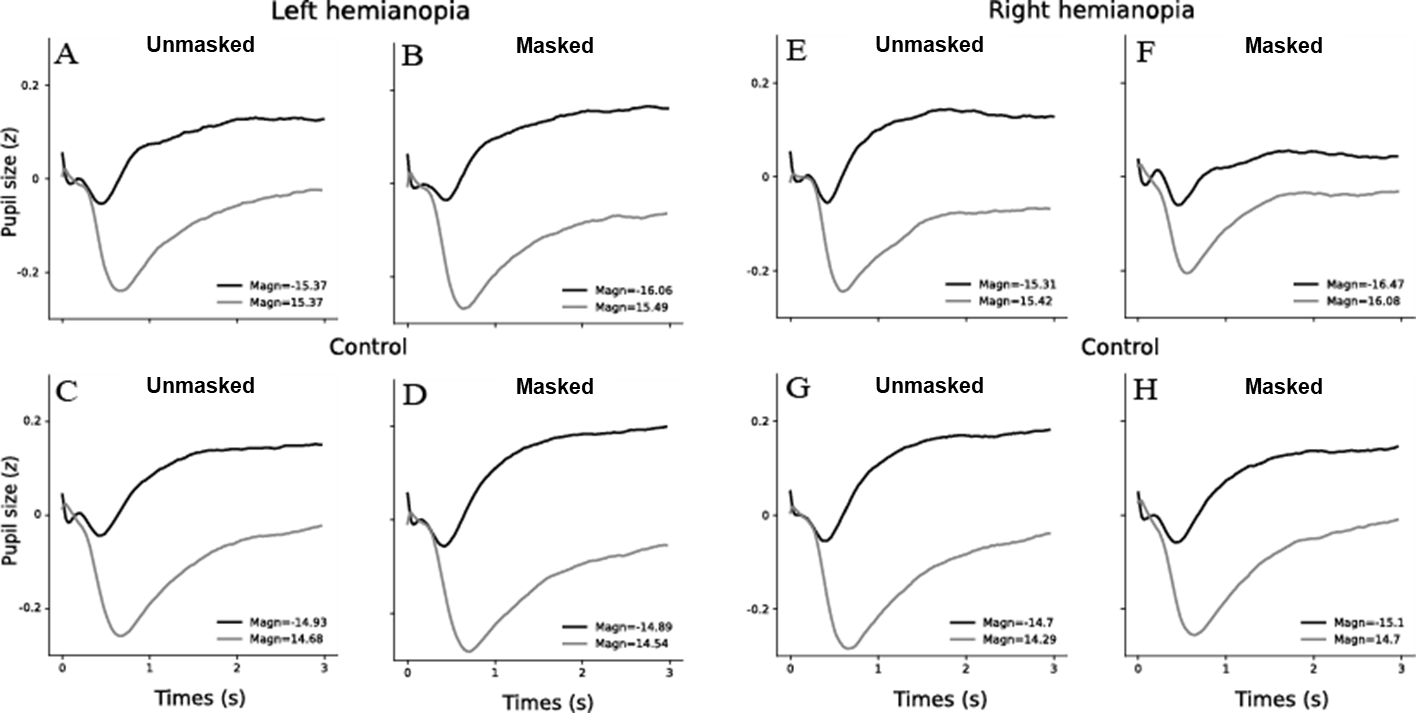


**Supplementary Fig. 3** Event-related pupil responses (ERPRs) to binned luminance changes. **A** Average ERPRs for unmasked regions and **B** for masked regions in simulated left hemianopia; **C-D** Average ERPRs for unmasked and masked regions in control trials for the same participants as A-B. The dark line indicates averaged pupil response to strongest negative luminance changes (bin0) and the light line indicates that of strongest positive luminance changes (bin4). **E-H** Same plots as A-D but for simulated right hemianopia.


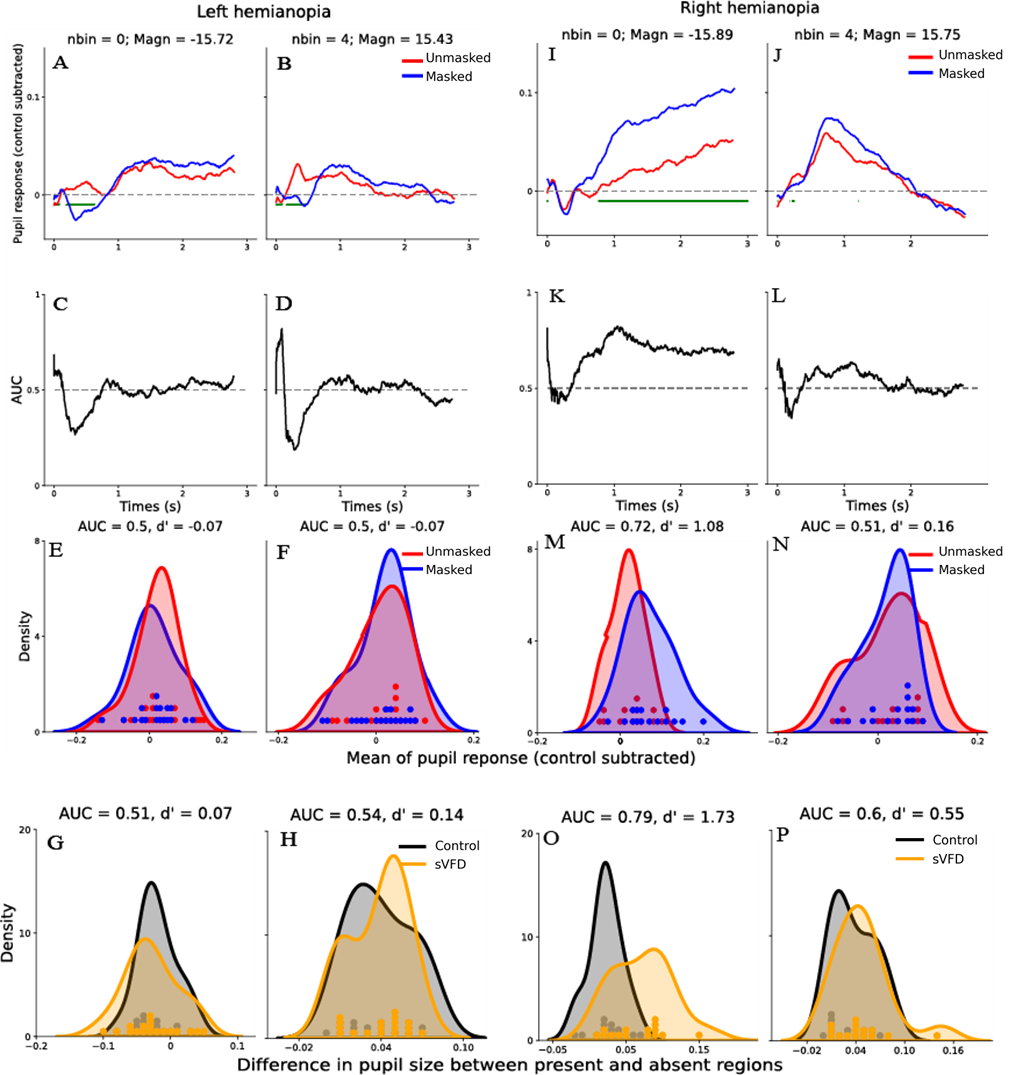


**Supplementary Fig. 4** **A-B** Control-subtracted pupillary responses for simulated left hemianopia for strongest negative (A, bin0) and positive luminance changes (B, bin4). Red lines indicate control-subtracted pupillary responses for unmasked regions and blue lines for masked regions. The four lines were calculated by the subtractions of lines in supplementary Fig. 3. For instance, the red line in A was the difference between the two dark grey lines in supplementary Fig.3 A and C. For negative luminance changes (bin4), the control-subtracted pupillary responses were further multiplied by -1. Green lines indicate the time points with a significant p-value for the paired-sample t-test between the pupil response for masked and unmasked regions (see main text for more details). **C-D** AUC values between the control-subtracted ERPR in unmasked and masked regions of simulated left hemianopia (two lines in A and B) for each of the time points, respectively for bin0 (C) and bin4 (D). **E-F** Distribution of the control-subtracted pupil response in unmasked (in red) and masked regions (in blue) of simulated left hemianopia after 0.5s for bin0 (E) and bin4 (F). **G-H** Distribution of difference between ERPRs in masked and unmasked regions for sVFD (orange) and control (grey) trials for simulated left hemianopia. **I-P** Same plots as A-D but for simulated right hemianopia


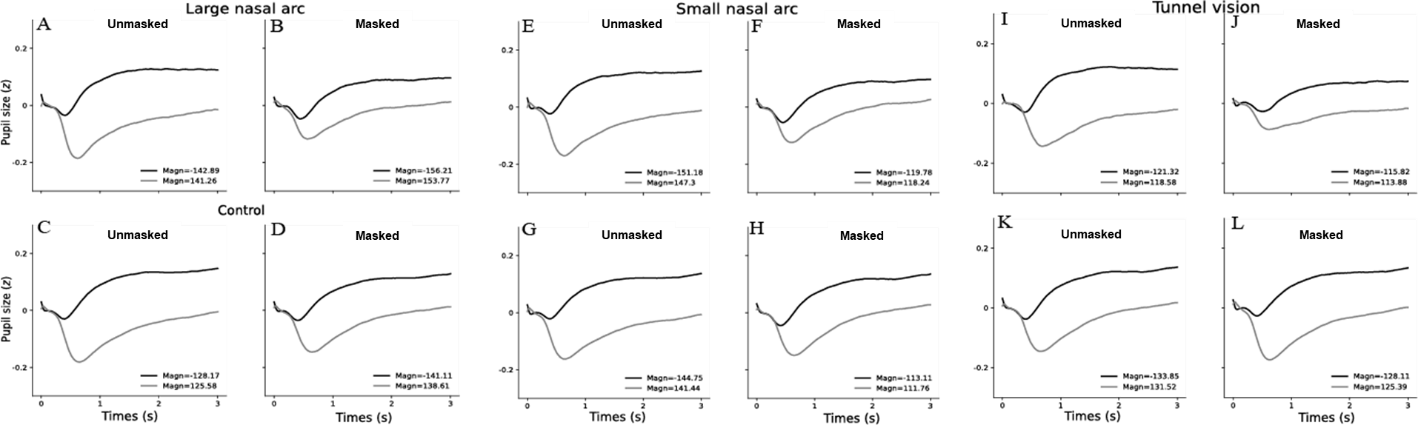


**Supplementary Fig. 5** Same plots as supplementary Fig. 3 but for simulated glaucoma. **A-D** Average ERPRs for simulated large nasal arc and controls; **E-H** Averaged ERPRs for simulated small nasal arc; **I-L** Averaged ERPRs for simulated tunnel vision.


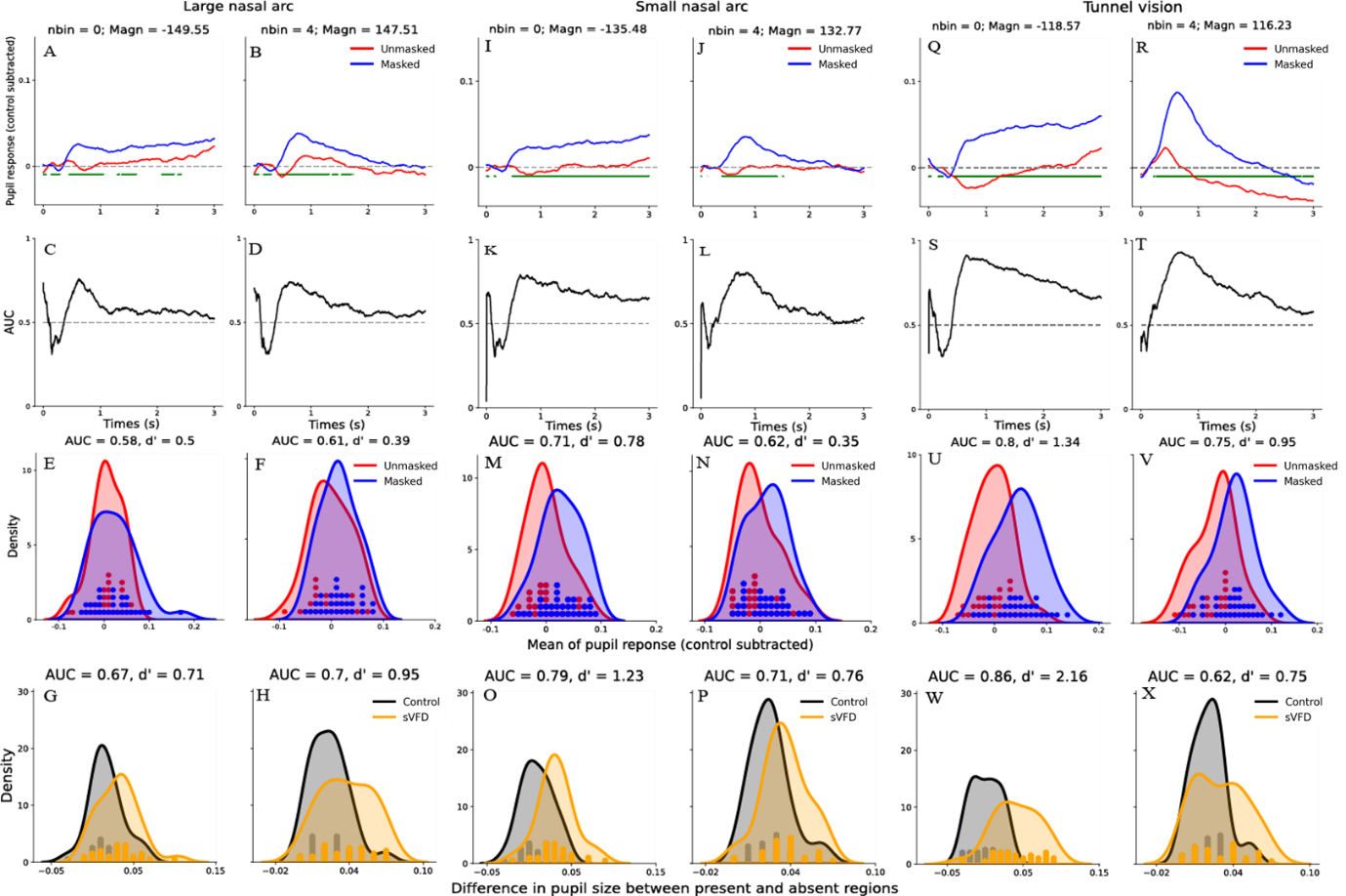


**Supplementary Fig. 6** Same plots as Fig. 4 but for simulated glaucoma. **A-H** For simulated large nasal arc; **I-P** For simulated small nasal arc; **Q-X** For simulated tunnel vision

## Results

### ERPR extraction

Pupil responses to strongest negative and positive luminance changes (bin0 and bin4) were depicted in **supplementary Fig. 3 and Fig. 5**. For simulated right hemianopia (**supplementary Fig. 3 E-F**) and three conditions of simulated glaucoma (**supplementary Fig. 5 A-B, E-F, I-J**), the pupil responses to events in masked regions were weaker than unmasked regions. However, for simulated left hemianopia, pupil response amplitudes failed to dissociate between masked and unmasked regions (**supplementary Fig. 3 A-B**).

### Control-subtracted pupil response

In line with the results reported above, results of a paired-sample t-test (**supplementary Fig. 4 and Fig. 6, first row, Green line**) illustrated that there were significant differences in control-subtracted pupil responses to luminance changes between unmasked and masked regions for simulated right hemianopia (**supplementary Fig. 4I**), and three conditions of simulated glaucoma (**supplementary Fig. 6 A-B, I-J, Q-R**). However, event related pupil responses to luminance changes could not be used to differentiate between masked and unmasked regions for simulated left hemianopia (**supplementary Fig. 4 A-B**).

### Differences in ERPRs between unmasked regions and masked regions

AUC values of the control-subtracted ERPRs between unmasked and masked regions for each time point were plotted in **supplementary Fig. 4 and Fig. 6, second row**, and the distribution of average control-subtracted pupil response between masked and unmasked regions was plotted in the same plots, third row. Control-subtracted ERPRs were larger for masked regions than unmasked regions for all conditions except simulated left hemianopia, with a highest AUC of 0.72 and 0.8, and a highest effect size of d’ 1.08 and 1.34 for simulated hemianopia and simulated glaucoma respectively (see **supplementary Fig. 4 and Fig. 6, third row** for the results of AUC and d’ in each condition).

### Sensitivity and accuracy of ERPRs in separating sVFD and control conditions

For each participant, the difference in ERPRs to luminance change between unmasked and masked regions under each sVFD condition for each sVFD condition was plotted with the corresponding control condition in **supplementary Fig. 4 and Fig. 6, fourth row.** The pupil response distributions for sVFD conditions and control conditions were fairly distinct, with a highest AUC of 0.79 and 0.86, a highest of d’ of 1.73 and 1.23 (see **supplementary Fig. 4 and Fig. 6, fourth row** for the results of AUC and d’ per condition).

## Discussion

The ERPR approach can be understood as a simplified version of the modeling approach and the idea underlying the two methods is essentially the same. However, the ERPR analysis had to first segregate the luminance changes into different levels and could not model continuous data as the modeling method. In addition, it cannot account for the covariance of luminance changes across visual field regions (i.e., global luminance changes resulted in similar luminance changes in masked and unmasked regions), which was likely to hamper its discriminative power. Also, event-related pupil responses can be disturbed or even canceled out when consecutive luminance changes have opposite signs.

1. For simulated hemianopia, as the numbers of masked and unmasked regions were the same, the average magnitude of luminance changes were used while for simulated glaucoma, as the number of masked and unmasked regions were not the same, the sum magnitude of luminance changes were used instead. [↑](#footnote-ref-2)
